# Treatment of recurrent renal transplant lithiasis: analysis of our experience and review of the relevant literature

**DOI:** 10.1186/s12882-020-01896-5

**Published:** 2020-06-23

**Authors:** Xiaohang Li, Baifeng Li, Yiman Meng, Lei Yang, Gang Wu, Hongwei Jing, Jianbin Bi, Jialin Zhang

**Affiliations:** 1 Department of Hepatobiliary Surgery, First Affiliated Hospital, China Medical University, No.155, Nanjing North Street, Heping District, Shenyang, 110001 Liaoning Province People’s Republic of China; 2 Department of Urology, First Affiliated Hospital, China Medical University, No.155, Nanjing North Street, Shenyang, 110001 Liaoning Province People’s Republic of China

**Keywords:** Renal transplant lithiasis, Transplanted kidney stone, Calculus, Recurrence, Treatment

## Abstract

**Background:**

Renal transplant lithiasis is a rather unusual disease, and the recurrence of lithiasis presents a challenging situation.

**Methods:**

We retrospectively analyzed the medical history of one patient who suffered renal transplant lithiasis twice, reviewed the relevant literature, and summarized the characteristics of this disease.

**Results:**

We retrieved 29 relevant studies with an incidence of 0.34 to 3.26% for renal transplant lithiasis. The summarized incidence was 0.52%, and the recurrence rate was 0.082%. The mean interval after transplantation was 33.43 ± 56.70 mo. Most of the patients (28.90%) were asymptomatic. The management included percutaneous nephrolithotripsy (PCNL, 22.10%), ureteroscope (URS, 22.65%), extracorporeal shockwave lithotripsy (ESWL, 18.60%) and conservative treatment (17.13%). In our case, the patient suffered from renal transplant lithiasis at 6 years posttransplantation, and the lithiasis recurred 16 months later. He presented oliguria, infection or acute renal failure (ARF) during the two attacks but without pain. PCNL along with URS and holmium laser lithotripsy were performed. The patient recovered well after surgery, except for a 3 mm residual stone in the calyx after the second surgery. He had normal renal function without any symptoms and was discharged with oral anticalculus drugs and strict follow-up at the clinic. Fortunately, the calculus passed spontaneously about 1 month later.

**Conclusions:**

Due to the lack of specific symptoms in the early stage, patients with renal transplant lithiasis may have delayed diagnosis and present ARF. Minimally invasive treatment is optimal, and the combined usage of two or more procedures is beneficial for patients. After surgery, taking anticalculus drugs, correcting metabolic disorders and avoiding UIT are key measures to prevent the recurrence of lithiasis.

## Background

Although renal transplant lithiasis is a rather unusual complication with an incidence of 0.2–1.7% [1], we should not underestimate its severity since it can lead to disastrous consequences, such as the loss of allograft, without timely treatment [2]. Renal transplant recipients usually need to attend regular follow-up after transplantation for vigilant monitoring of allograft function. Therefore, sometimes renal transplant lithiasis may be diagnosed during follow-up without symptoms. Early-stage renal transplant lithiasis has less influence on allograft function. Unlike common kidney lithiasis, renal transplant lithiasis patients may not present obvious pain due to denervation of the allograft kidney [3]. Consequently, some severe renal transplant lithiasis patients without regular follow-up may not be diagnosed until they present hydronephrosis, fever, or acute renal failure (ARF). In this situation, emergent treatment is necessary to avoid loss of allograft.

In this study, we described a patient experiencing renal transplant lithiasis twice and the detailed process of attack, diagnosis and treatment. We also reviewed the relevant literature, analyzing the patient characteristics, predisposing factors, symptoms, therapy, complications and outcomes, and compared them with our case to obtain a better understanding of this disease. As renal transplant recipients have relatively lower levels of immunity, delays in diagnosis and treatment may result in severe infection. All physicians in this field should be alert to this rare but potentially deadly disease to provide patients with optimal management.

## Methods

We collected the detailed data of our case with renal transplant lithiasis, including date of transplantation, antirejective medication, clinical presentation, laboratory tests, imaging examination, therapy, and another process of diagnosis and treatment after recurrence. Two electronic databases (PubMed and Web of Science) were searched for relevant clinical studies and case series, excluding case reports and reviews, published in English from 2000 to 2019. The following keywords were used for the literature search: “renal transplant lithiasis / calculus” or “kidney transplant lithiasis / calculus” or “ureteral calculus / stone” or “urinary tract calculus” or “recurrence of lithiasis” or “recurrent lithiasis,” in combination with “kidney / renal transplantation”. The reference lists of these articles retrieved online were reviewed for potentially eligible studies. We extracted the data from all the relevant articles; summarized the patients’ demography and symptoms; analyzed stone characteristics, treatment strategies, complications and outcomes; and then compared them with our patient’s. Therefore, we can summarize the experience of management in detail. We don’t have adhered to PRISMA and the intention of the literature review was to summarize the related literature in order to determine whether the outcomes of our case comply with current beliefs about the problem.

## Results

### Case presentation

**Fig. 1:**
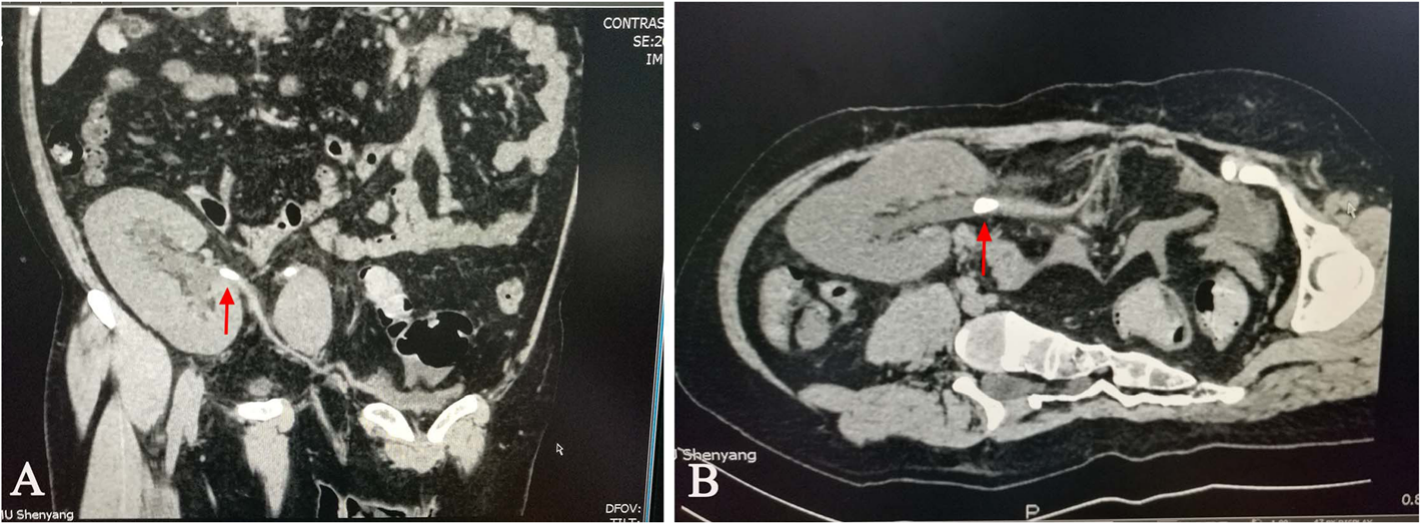
Preoperative CT scan showing a ureteral calculus in the ureteropelvic junction. **a** Coronal plane, **b** transverse plane

**Fig. 2:**
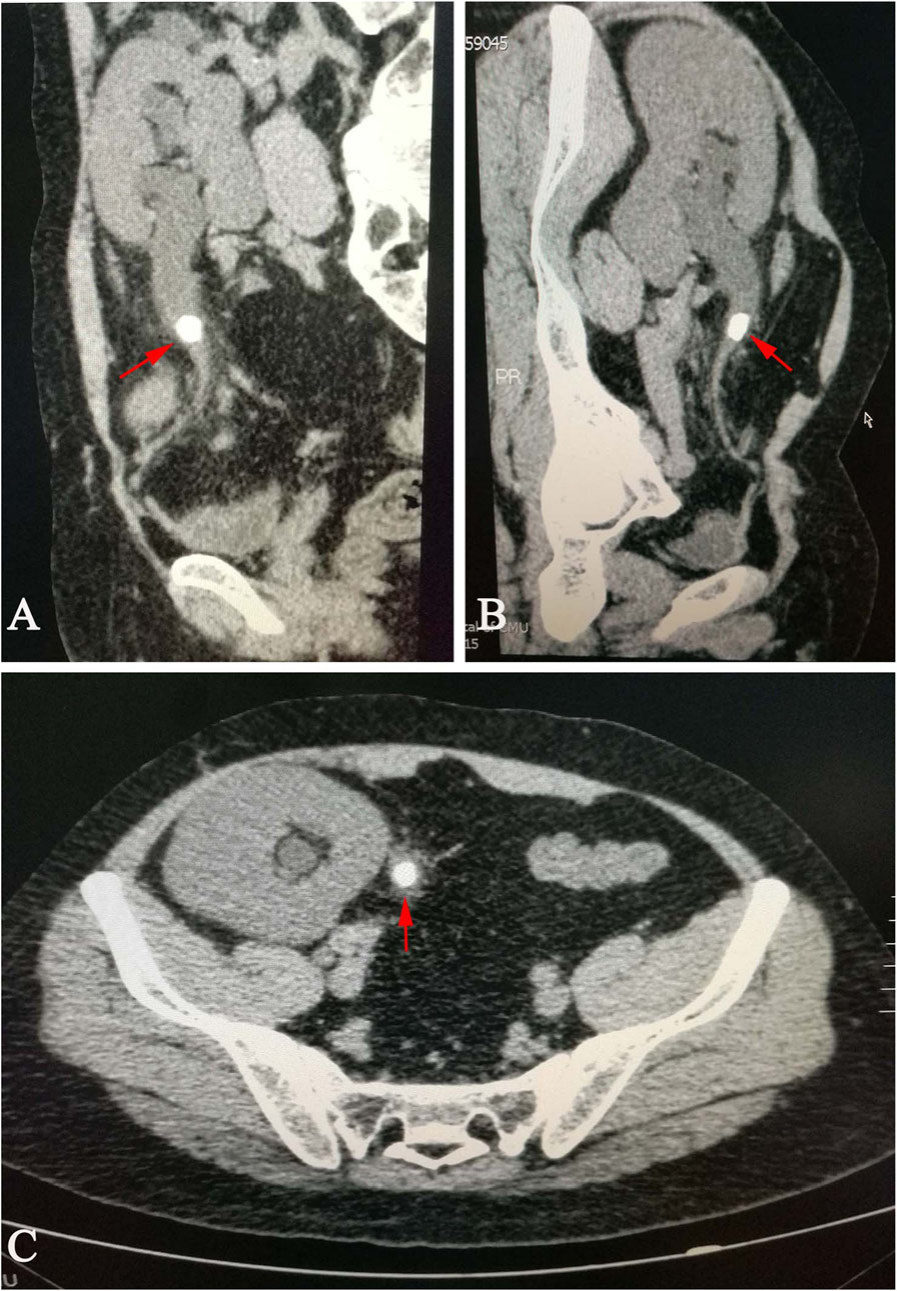
Preoperative CT scan showing a proximal ureteral calculus with severe hydronephrosis and extension of the ureter. **a** Longitudinal plane left view, **b** longitudinal plane right view, **c** transverse plane

**Fig. 3:**
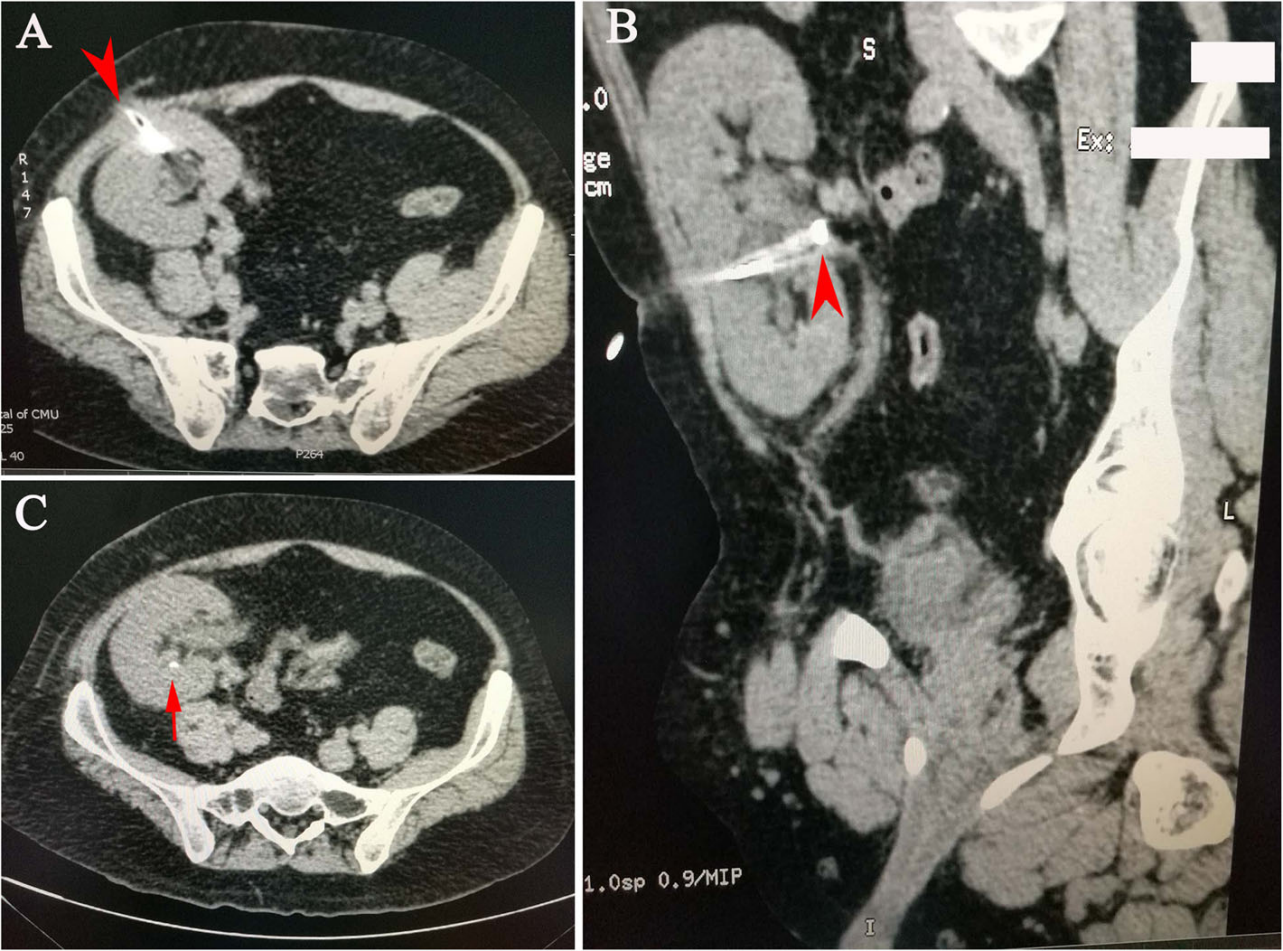
Postoperative CT scan showing a residual calculus in the lateral calyx. **a** Transverse plane. The red arrowhead shows the percutaneous nephrostomy tube, **b** Longitudinal plane. The red arrowhead shows the percutaneous nephrostomy tube, **c** Transverse plane. The red arrow shows the residual calculus

A 35-year-old male with end-stage renal disease of unknown cause underwent cadaveric renal transplantation in our department 6 years ago. Due to a lack of kidney biopsy, we did not know the exact etiology causing him to develop an end-stage renal disease. The postoperative immunosuppression regimen consisted of tacrolimus, mycophenolate mofetil and prednisolone. The patient was followed at another clinic at approximately 8-month intervals. There was no obvious abnormality except for medium microscopic haematuria for his latest follow-up. Unfortunately, the doctor in that clinic did not recommend further examination, so we had no idea about the source or morphology of the red blood cells in urine. The patient did not have a history of hypertension, diabetes or hyperparathyroidism. He had a desk job, so he might have been too sedentary. He did not have a bad lifestyle, such as drinking alcohol or smoking, except for drinking Coca-Cola (1 can per day) for 3 to 4 years. On the 6th year after transplantation, the patient suddenly fevered with a temperature of 38.8 °C and shiver, accompanied by oliguria with 400–500 mL urine/d and little gross haematuria. He was admitted to emergency department. Physical examination indicated a heart rate (HR) of 92 bpm, blood pressure (BP) of 131/88 mmHg, Body Mass Index (BMI) of 27.5 kg/m^2^, and mild tenderness in the graft area. Blood chemical test showed a white blood cell count (WBC) of 7.37 × 10^9^/L, lymphocytes 11%, neutrophils 79%, serum creatinine 4.04 mg/dL, blood glucose 109.8 mg/dL, serum uric acid 6.4 mg/dL, blood calcium 8.90 mg/dL, serum albumin 2.73 g/dL, and corrected calcium 9.90 mg/dL. The pH value of the urine was 6.2. Computerized tomography (CT) showed that there was a stone with a size of 18 mm in the ureteropelvic junction, which caused mild hydronephrosis (Fig. 1). An emergent operation was arranged in combination with efficient antimicrobial therapy. A ureteroscope (URS) was performed in the lithotomy position under general anesthesia. We could not insert the guide wire into the new orifice with a 70° lens ureteroscope, although we switched to a semirigid ureteroscope. Therefore, we had to perform percutaneous nephrolithotripsy (PCNL) to remove the stones. The anterior calyx in the upper pole was chosen for puncture under ultrasonography guidance. An nephroscope was inserted through the sheath to inspect the pelvicalyceal system, and a 9.5–10 Fr flexible URS was used to inspect the ureter. A brown stone of 18 mm was located in the ureteropelvic junction. The stone was fragmented and extracted. After the stone was confirmed free by intraoperative ultrasonography, a 4.8 Fr double-pigtail stent was introduced. A 14 Fr nephrostomy tube was placed. After operation, the patient’s condition improved quickly, with normal temperature and gradually increased urine (2300 mL urine/day), and serum creatinine decreased to 1.65 mg/dL. The nephrostomy tube was removed 3 days later. The patient was discharged 10 days postoperation with normal urine and serum creatinine, and a stone-free condition was confirmed by ultrasonography. A double-pigtail stent was removed 4 weeks later. After discharge, the patient was followed up every 3 months and then every 6 months 1 year later. There were no abnormalities during follow-up. Unexpectedly, the patient was admitted to the hospital again 16 months after the operation for similar symptoms, including fever (39 °C), shivers, and anuria, but without pain. Blood chemical tests indicated severe infection and ARF (WBC 8.14 × 10^9^/L, lymphocytes 11.2%, neutrophils 84.2%, and serum creatine 6.68 mg/dL), blood glucose was 113.4 mg/dL, serum uric acid was 6.6 mg/dL, blood calcium was 8.82 mg/dL, serum albumin was 2.55 g/dL, and corrected calcium was 10.0 mg/dL. The pH value of the urine was 5.7. CT demonstrated a 12 mm calculus in the proximal ureter with severe extension of the ureter and hydronephrosis (Fig. 2). A 14 Fr percutaneous nephrostomy tube was first placed emergently to promptly decompress and relieve the symptoms; in this case, an interpolar middle calyx was accessed. The patient gradually recovered, with normal temperature and approximately 1800 mL urine/day through the nephrostomy tube, and serum creatinine decreased to 3.58 mg/dL. After the improvement of allograft function, PCNL was performed. The nephroscope and flexible URS were advanced into the pelvicalyceal system or ureter through the existing access tract. Holmium laser lithotripsy was performed to fragment the yellow-brown solid calculus located in the ureter, and fragments were completely removed. After confirming that all stones were taken out, a 16 Fr nephrostomy tube was placed, without introducing a double-pigtail stent. After the operation, allograft function was further improved, with urine 1500 mL/day and serum creatine of 1.33 mg/dL. The percutaneous nephrostomy tube was removed 4 days later. Analysis of stone composition indicated a uric acid calculus, so potassium sodium hydrogen citrate (2.5 g, 3 times a day) was administered. On the 19th day postoperation, serum creatine decreased to 1.07 mg/dL. Ultrasonography indicated hyperechogenicity in the kidney. Therefore, CT was performed again and indicated that there was a small residual stone with a size of 3 mm in the calyx (Fig. 3). The patient had normal urine and renal function without pain or haematuria, and he was discharged with strict follow-up in the clinic. As the BMI of the patient was slightly high, we recommended that he lose some weight, do some exercise, and drink more water instead of Coca-Cola. Fortunately, the calculus passed through the urine tract spontaneously about 1 month later. Now the patient is healthy with normal allograft function and free of renal transplant lithiasis.

### Characteristics of the retrieved relevant studies

**Fig. 4:**
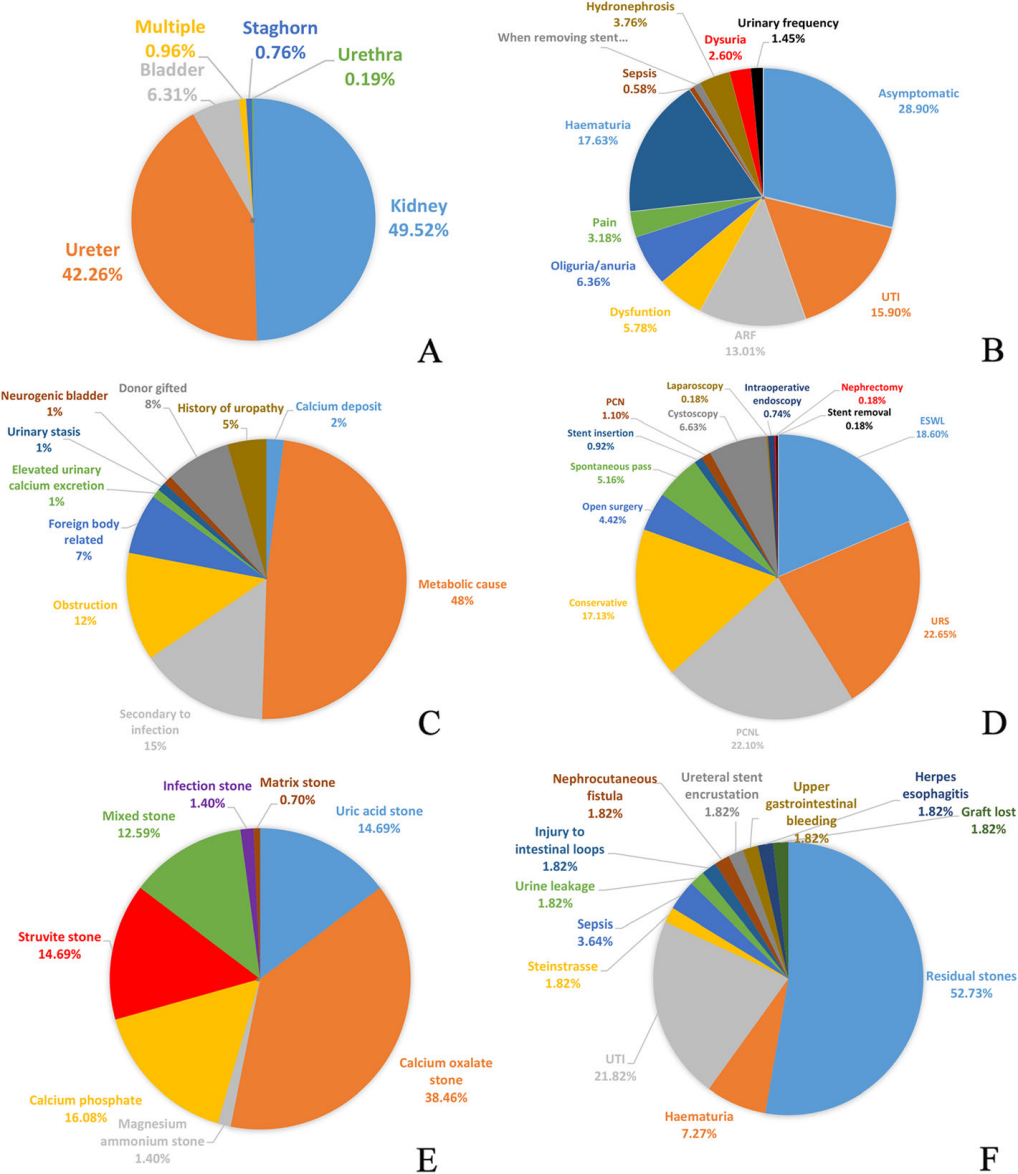
Characteristics of renal transplantation lithiasis and management. **a** The distribution of stone locations, **b** the clinical presentation of patients with renal transplant lithiasis, **c** the etiologies of renal transplant lithiasis, **d** the management of patients with renal transplant lithiasis, **e** the composition of transplanted kidney stones,and **f** the complications after operation

Twenty-nine relevant studies were retrieved and analyzed [4–32], and the characteristics of these studies are listed in Additional file 1. The incidence of renal transplant lithiasis was between 0.34 and 3.26%. There were 551 cases with renal calculus, except for 29 cases of bladder calculus and 1 case of urethra calculus. The number of renal transplant recipients included in these studies ranged between 125 and 42,096. After excluding the studies with missing numbers of recipients [5, 12, 13, 17, 18, 21, 24, 30], the summarized renal transplant lithiasis accidence was 0.52%. A total of 25 patients with recurrent renal transplant lithiasis after operation was reported in 13 studies [6, 8–10, 14, 15, 17, 20–22, 24, 26, 30], and the recurrence rate was 0.082%. The mean interval after transplantation was 33.43 ± 56.70 mo. Figure 4a shows the distribution of stone locations. The patients with renal transplant lithiasis presented many kinds of clinical manifestations (Fig. 4b), but asymptomatic patients accounted for the largest proportion (28.90%). Along with donor-gifted stones (7.5%), the common etiologies of calculus are metabolic disorder (48.5%), infection (15%) and urinary obstruction (12.5%) (Fig. 4c). The management included PCNL (22.10%), URS (22.65%), extracorporeal shockwave lithotripsy (ESWL,18.60%) and conservative treatment (17.13%)(Fig. 4d). We also noted that stones passed spontaneously in 5.16% of patients, and open surgeries were performed only in 4.42% of patients. Twelve studies reported that the stone composition was analyzed (Fig. 4e). The most common calculi were calcium-based [54.54%: calcium oxalate stones (38.46%), calcium phosphate stones (16.08%)], followed by uric acid stones (14.69%), struvite stones (14.69%), mixed stones (12.59%), magnesium ammonium stones (1.40%), infection stones (1.40%), matrix stones (0.70%). Fifty-five patients with complications after operation were reported in 24 studies (Fig. 4f). The most frequent complication was residual stones (52.73%), followed by urinary tract infection (UTI, 21.82%), haematuria (7.27%) and sepsis (3.64%). Only one patient (1.82%) had to receive transplanted nephrectomy due to the serious illness caused by the renal calculus.

## Discussion and conclusions

According to the relevant studies retrieved in the databases, the incidence of renal transplant lithiasis is low (0.52%), and it is the least described urologic complication of renal transplantation. However, urinary obstruction caused by renal transplant lithiasis can lead to a devastating loss of allograft without timely and appropriate therapy. We should pay more attention to this complication.

It seems that there is no obvious etiology for the patient reported in this article, but his overweight and excessive consumption of Coca-cola maybe major risk factors. His symptoms were not specific and therapy method was effective. Unfortunately, recurrent lithiasis was diagnosed for this patient 16 months after the first operation. How to treat the recurrent renal transplant lithiasis is another import point.

The etiology of renal transplant lithiasis includes donor grafting, metabolic abnormalities and mechanical factors. Renal transplant lithiasis identified within 6 weeks after transplantation is usually accepted as native to the donor kidney [33]. The most common metabolic abnormalities contributing to renal calculus are hyperparathyroidism and hyperuricemia. Hyperparathyroidism usually exists in patients with uremia due to systemic metabolic abnormalities. It leads to hypercalcemia, which is one predisposing factor of renal calculus. Many patients with uremia even suffer from native kidney stones before transplantation, although they are usually asymptomatic. Our patient had been on dialysis treatment for 3 years before transplantation, but he did not present native kidney stones. Most recipients present with hyperuricemia after kidney transplantation, which is one of the side effects of immunosuppressive drugs. Norlen et al. considered that cyclosporine produced hyperuricosuria in approximately 50 to 60% of patients receiving this medication for immunosuppression [34]. Ureteral stricture or obstruction can lead to renal transplant lithiasis. Patients with UTIs are more likely to suffer from renal stones, and renal stones can lead to UTIs.

Unexpectedly, our patient did not have any predisposing factors mentioned above, and only the corrected calcium level reached the upper limit of normal. However, we noticed that his BMI was 27.5 kg/m^2^, meaning he was in the overweight range. Taylor et al. considered that overweight and obesity increase the risk of kidney stone formation [35]. Curhan GC also confirmed that a higher BMI is associated with a higher risk of kidney stones [36]. A large prospective cohort study demonstrated that the strongest risk factor associated with a high risk of incident kidney stones was a higher BMI [37]. This patient also drank Coca-Cola (1 can per day) for 3 to 4 years prior to his first hospital admission. Ferraro et al. reported that more frequent consumption of sugar-sweetened beverages was associated with a 30–40% higher risk of kidney stones [38]. This patient also did not move his body enough. Sorense MD found that physical activity can reduce the risk of incident kidney stones in postmenopausal women [39]. Therefore, this patient’s diet and lifestyle may have contributed to the formation and recurrence of renal transplant lithiasis.

Regarding immunosuppressive medications, this patient was taking tacrolimus (2.5 mg, BID), mycophenolate mofetil (750 mg, BID) and prednisolone (5 mg, QD) at his first admission. Kanbay et al. demonstrated that compared with cyclosporine, tacrolimus offers no advantage for serum uric acid level in renal transplant recipients [40]. Mycophenolate mofetil seldom causes hyperuricemia [41]. Liu C demonstrated that low-dose (15 mg/day) and medium-dose (30 mg/day) prednisone had no effect on serum uric acid level [42]. His tacrolimus blood level ranged from 4.10 to 9.60 ng/ml. Therefore, we think that tacrolimus might not have played an important role in the formation or recurrence of his stones. We did not analyze the stone composition after the first operation. Based on the constituent of his calculus after the second operation, we deduced that the level of uric acid in the urine may have been higher, although it was normal in the blood. Unfortunately, we did not detect uric acid in urine. In the future, when we collect more patients, we will test more items to better analyze the etiology.

The patients with renal transplant lithiasis usually have no specific symptoms. They can present with oliguria, anuria, haematuria, frequent urination, UTI, allograft dysfunction or even ARF, but seldom with graft pain. Our data also demonstrated that only 3.18% of patients presented pain, and 28.90% of them were asymptomatic. Therefore, the patient usually has a high propensity for a delayed diagnosis. Our patient presented oliguria, anuria, UTI and ARF during the two attacks, but without pain. Due to lack of early manifestations, he was not diagnosed until the advanced stages of the disease.

Management options for renal calculus include conservative treatment, ESWL, PCNL, URS and open surgery, and the choice of treatments depends on the patient’s symptoms, etiology, and position and size of the stone. For patients with stones less than 4 mm and presenting no symptoms or urinary obstruction, it is more likely for stones to pass spontaneously, and conservative treatment can be chosen with close follow-up [16]. An alkalinizing drug can be recommended for patients with radiolucent stones and lower-pH urine during follow-up. ESWL is recommended for patients with stones less than 15 mm [43], and PCNL is widely used for renal transplant lithiasis patients with stones larger than 2 cm or when ESWL fails [27]. The serious perinephric fibrosis caused by immunosuppressive drugs could increase the difficulty of puncturing and risks of bleeding [21]. When multiple operations are needed, we should choose different accesses for puncturing to decrease the risk of bleeding. URS can be performed without any incision in the body. It is appropriate for ureteral stones. Nevertheless, it is difficult to insert a guide wire into the transplanted ureter through a new orifice. This was found in our case. In this situation, a semirigid ureteroscope or a 70° lens can be used to facilitate the process. Some articles reported a 60–67% success rate for extracting a ureteral stone by URS [29, 44]. URS can synergize with holmium laser lithotripsy to completely remove a calculus. With the development of endoscopic techniques for the management of urological lithiasis, the importance of open surgery is gradually decreasing. From the retrieved literature, we can see that the rate of open surgery is very low (4.35%). Now open surgery is considered only when patients burdened by ureteral stricture or giant staghorn calculi cannot be cured by other management methods.

Based on our data, the most frequent complication was residual stones (52.73%). Nephroscope or URS in combination with intraoperative ultrasonography should be used to exclude residual stones after the operation. If it is impossible to remove all the stones, a small stone less than 4 mm can be left with close follow-up after operation. After removal of the stones, components of the stones should be tested to guide pharmaceutical treatment to avoid recurrence of lithiasis. We think the most common reason for recurrence of lithiasis is exposure to predisposing factors that are not reduced or eliminated after surgery. Repeated UTI after surgery and retained foreign body, such as stent and prolene suture, also contribute to the recurrence of lithiasis. Ureteral stents are usually placed at the end of ureteroscopic management [45]. Although Branchereau et al. considered that stents are not a risk factor for early stone formation [5], we still suggest that patients with stents have a higher propensity to develop renal stones than those without stents. Therefore, we did not place a stent after the second operation. Correcting metabolic disorders, treating UTIs and removing stents in a timely manner after surgery are pivotal to prevent the recurrence of lithiasis.

To our best knowledge, few articles have reported treatment protocols for the recurrence of lithiasis in transplanted kidneys. We think it should mimic the protocol that was performed for them during their first attack. Minimally invasive surgery is optimal if conserved treatment is not effective. A strong effort to remove all the stones and preventive drug therapy after operation are suggested, according to the composition of the stone. A close follow-up composed of renal ultrasonography, urinalysis and renal function is necessary. We will collect more cases to statistically analyze the risks for recurrence of renal transplant lithiasis in the future.

Renal transplant lithiasis is unusual and special, as recipients taking immunosuppressive drugs are liable to infect. We should note that these patients usually have delayed diagnosis and even present ARF as an initial symptom since they usually do not have symptoms of graft pain. Based on the patients’ characteristics, the combined usage of two or more minimally invasive procedures is beneficial to improve the efficiency and promote recovery after surgery. Patients should be rendered stone free at the end of the procedure. After surgery, taking anticalculus drugs, correcting metabolic disorders and avoiding UTIs are key measures to prevent the recurrence of lithiasis. The patients are recommended to cooperate with the close follow-up and develop a healthy lifestyle with enough water intake and moderate exercise, especially those with predisposing factors.

## Supplementary information

**Additional file 1: Table 1.** Characteristics of the retrieved relevant studies.

## Data Availability

All data generated or analysed during this study are available from the corresponding author on reasonable request.

## Abbreviations

ARF: Acute renal failure
UTI: Urinary tract infection
ESWL: Extracorporeal shockwave lithotripsy
PCNL: Percutaneous nephrolithotomy
URS: Ureteroscope
PCN: Percutaneous nephrostomy
BPH: Benign prostate hyperplasia
