# Supplementary material for: Treatment of recurrent renal transplant lithiasis: analysis of our experience and review of the relevant literature
Source: BMC Nephrol. 2020 Jun 23;21:238. doi: 10.1186/s12882-020-01896-5 (PMC7310338; doi:10.1186/s12882-020-01896-5)
Supplement: Supplementary file 1 — Additional file 1: Table 1. Characteristics of the retrieved relevant studies. [file 12882_2020_1896_MOESM1_ESM.docx]

**Table 1.** Characteristics of the retrieved relevant studies

| Author | Country | Year | Recipient Number | During | Patients of lithiasis | Incidence of lithiasis(%) | Age:mean(range)(y) | Gender(male/female) | Cadaveric/ Living donor | Stonesize :mean(range) (mm) | Stone locatioin | Interval after transplantation:mean(range) | Symptom | Etiology | Management | Composition of stone | Complication | Recurrence during follow-up |
| --- | --- | --- | --- | --- | --- | --- | --- | --- | --- | --- | --- | --- | --- | --- | --- | --- | --- | --- |
| Abbott ^4^ | USA | 2003 | 42096 | 1994.7.1-1998.6.30 | 52 | 1.23% | NA | NA | NA | NA | Kidney 35, ureter 17 | NA | NA | NA | ESWL1，URS 7，PCNL 19 | NA | NA | NA |
| Branchereau ^5^ | France | 2018 | NA | NA | 95 | NA | 48 | 63/32 | NA | 9 | Kidney 51, ureter 44 | 40.5m | Asymptomatic 52,UTI 21,ARF 22 | Hyperuricaemia 6，hyperparathyroidism 11， calcium  deposit 4，a history of uropathy 9，donor gifted 7 | Conservative treatment 46,ESWL12,URS 25,PCNL10,open surgery 2,11residual(9 conservative treatment+2 PCNL) | NA | Residual stones 9 | No recurrence |
| Challacombe ^6^ | UK | 2005 | 2085 | 1997-2003 | 21 | 0.96%b | 41(45-64) | 8/13 | 18/3 | 8.1(4–17) | Kidney 13,ureter 7,bladder 1 | 3.6(0.5-18)y | Oliguria or anuria 6(1ARF), pain 4, hematuria 3, sepsis 1, when removing a stent 1, asymptomatic 6 | Metabolic cause 10,obstruction 2,stent-related 2,secondary to infection 1 | ESWL(8 multiple sessions) 13,Stent insertion 5, PCN 3, URS 2, PCNL 3, open surgery 2, spontaneous pass 2 | NA | Residual stone 1 | Recurrence 1 |
| Cicerello ^7^ | Italy | 2014 | 953 | 1995.1-2012.12 | 10 | 1.05% | 43(31-59) | 4/6 | NA | 12(7-30) | Kidney 7,ureter 3 | NA | Asymptomatic 6, oliguria 1, anuria and ARF 1, at removal of the ureteral stent 2 | Hyperparathyroidism 5,  hyperuricaemia 3, secondary to infection 4 | ESWL 2, PCNL 2,URS 5,Open surgery 1 | NA | NA | NA |
| Doehn ^8^ | Germany | 2002 | 1500 | 1985-2000 | 11 | 0.73% | 50^a^ | 5/6 | 11/0 | NA | NA | NA | Hematuria 9, hydronephrosis and dysfunction 4, UTI 3 | NA | ESWL 3, URS 2,PCNL 3, conservative treatment 3 | Uric acid stone 3, calcium oxalate stone3;magnesium ammonium stone 2 | No complication | Recurrence 2 |
| Emiliani ^9^ | Italy | 2018 | 2115 | 1983-2017 | 51 | 2.41% | 48.9 (26-70) | 39/12 | 45/6 | 9(2-30) | Kidney 19, ureter 32 | 30.5^a^(10–63)m | ARF 12, UTI 3, hematuria 12, pain 2, asymptomatic 22 | NA | ESWL 22, PCNL 2,URS 11, open surgery 2, conservative treatment 14 | Calcium  oxalate stone7, calcium phosphate stone 4, calcium  oxalate stone 1,uric acid stone 6, struvite stone 5,mixed stone 1 | Hematuria 2， UTI 3, steinstrasse 1,sepsis 1; residual stones 15 | Recurrence 4 |
| Ferreira ^10^ | Brazil | 2012 | 1313 | 1968.2-2011.2 | 17 | 1.29% | 45.6(32-63) | 8/9 | 15/2 | 7.4(2-15) | Kidney 12, ureter 5 | During operation 2;<7 day3；6m-13y: 12 | UTI 2, dysfunction 3 | NA | Conservative treatment 3, open surgery 2,ESWL 6,PCNL 1,URS 3,spontaneous pass 2 | NA | No complication | Recurrence 3 |
| Harrza ^11^ | Egypt | 2017 | 1208 | 1974-2009 | 16 | 1.08%b | 41 | 14/2 | 0/16 | 13.8 (5-40) | Kidney 13, ureter 3, bladder 6 | 170^a^ (51-351)m | NA | NA | Conservative treatment 3,ESWL 3, cystoscopy 7, PCN 3, PCNL 6 | NA | Hematuria 1, urine leakage 1,PCN-induced injury to the  intestinal loops 1, residual stone 2 | No recurrence |
| He ^12^ | China | 2007 | NA | 2002.8-2006.6 | 7 | NA | 40.7(28-54) | 5/2 | 5/2 | 17(6-40) | Staghorn 1, kidney 2,ureter 4 | 38.7m(1m-6y) | Asymptomatic 3,UIT 1,oliguria with dysfuntion 3 | Hyperuricaemia 3 | PCNL 7 | Calcium oxalate stone 2,uric acid stone 2,mixed stone 2,struvite stone 2 | No complication | No currence |
| Hyams ^13^ | USA/Canada | 201 | NA | 2006-2011 | 12 | NA | 42(36–72) | 6/6 | 5/7 | 8 (4–15) | Kidney 9,ureter 5(2patients had both ureteral and intrarenal stones) | 87(8–209) m | Hydronephrosis 6, UTI 3,pain 2, asymptomatic 1 | NA | URS 12 | Calcium oxalate stone 6, calcium phosphate stone 4, struvite stone 1 | Nephrocutaneous fistula 1,ureteral stent encrustation 1,residual stone 1 | No recurrence |
| Khositseth ^14^ | USA | 2004 | 399 | 1986.9-2003.1 | 20 | 3.26%b | 9.2 | 17/3 | 2/18 | NA | Kidney 1 ,  ureter 10 ,  Multiple sites 2, Bladder 7 | 19 (2-72)m | UTI 8, hematuria, 5, microscopic hematuria, 2; dysuria 9, asymptomatic 2 | suture retention 4, elevated urinary calcium excretion 2,  UTI 2, urinary stasis 2 | Cystoscopy 11 ,  Spontaneous pass 4 ,  Open surgery 3,  Laparoscopy 1 | Calcium phosphate stone 6 ,Calcium oxalate stone 2,  mixed stone 1,Struvite stone 2 | UTI 8 | Recurrence 5(including 1 bladder stone) |
| Kim ^15^ | Korea | 2001 | 849 | 1980-1997 | 15 | 0.47%b | 41.5(28-67) | 10/5 | 7/8 | 12(3.4-40) | Kidney 3,multiple sites 1, bladder 11 | 17.8(3-109)m | Hematuria 6, urinary frequency 5, asymptomatic 4 | Hyperparathyroidism 8, hypercalciuria 5, secondary to infection 5,  Hypocitraturia 4, obstructive uropathy 2. | Spontaneous pass 5, cystoscopy 8, conservative treatment 2 | Mixed stone 5,  calcium oxalate stone 1,calcium phosphate stone 3, Struvite stone 4 | No complication | Recurrence 5 |
| Klingler ^16^ | Austria | 2002 | 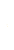1027 | 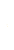1993.11-2000.12 | 19 | 1.85% | 48.1(26-72) | 8/11 | 18/1 | 11(4-47) | Kidney 19,ureter 3 | Intraoperative in 4, perioperative(12-25d) in 5,de novo(28:13-48m) in 10 | NA | Donor gifted 9 | Introperative endoscopy 4, ESWL 7, spontaneous pass 3, urteroscopy 1, PCNL 3, open surgery 1 | Calcium oxalate stone 11, Calcium phosphate stone 1, uric acid stone 2 | No complication | No currence |
| Krambeck ^17^ | USA | 2008 | NA | 1998.6-2006.2 | 13 | NA | 49.5(16-71) | 9/4 | NA | 13.6(2-30) | Kidney 1，ureter11,multiple 1 | 2.7(0.7-10)y | ARF 6 ,Hematuria 3,UIT3 ,Pain 2,sepsis 1 ,Hydronephrosis 1 | Neurogenic bladder 2,hyperparathyroidism 4,UTI 8 | PCNL 13 | Calcium oxalate stone 7，calcium phosphate stone 3，matrix stone 1，  struvite stone 1，  uric acid stone 1 | Sepsis1, upper gastrointestinal bleeding 1, herpes esophagitis 1 | Recurrence 1 |
| Li ^18^ | China | 2011 | NA | 1979-2009 | 10 | NA | 34(21-42) | 7/3 | 8/2 | 4-12 | Kidney 2,ureter 8 | 11.3(1-27)m | Acute oliguria or anuria and gas pain 3, hematuria 5, asymptomatic 2 | NA | ESWL 7, cystoscopy 2, open surgery 1 | NA | No complication | No recurrence. |
| Mahdavi ^19^ | Iran | 2014 | 1800 | 1989.1-2011.9 | 21 | 1.17% | 31 | 13/8 | 6/15 | 11 (6-18) | Kidney 11,  ureter 10 | NA | hematuria 5,  dysfuntion 4, anuria 3, urinary  tract infection 3, hydronephrosis 2 | Hyperparathyroidism 6, hyperuricaemia  5, urinary tract infection  4, ureteral stricture or obstruction 2 | ESWL 10(3 Stone unfree PCNL), URS 9(1 Stone unfree PCNL),PCNL 2 | NA | NA | NA |
| Mamarelis ^20^ | Greece | 2014 | 2045 | 1983.1-2013.7 | 7 | 0.34% | NA | NA | NA | 14.5(4-25) | Kidney 5, ureter:2 | 3.7(2-7) y | Hematuria 7,of them 2 dysfunction, 1 Oliguria | NA | PCNL 3, ESWL 3,spontaneous pass 1, | NA | NA | Recurrence 2 |
| Oliveira ^21^ | Portugal | 2010 | NA | 2002.4-2009.8 | 7 | NA | 44(28-60) | 3/4 | 6/1 | 32.8(20-50) | Kidney 4,staghorn 3 | 5.6(0.25-17.3)y | UTI(ARF2)5,hematuria 1,ARF 1 | Hyperuricaemia 2, hyperparathyroidism 2, secondary to infection 2,retained double-J ureteral stent 1 | PCNL 7 | NA | No complication | Recurrence 1 |
| Ozkaptan ^22^ | Turkey | 2018 | 920 | 2008-2015 | 7 | 0.76% | NA | NA | NA | NA | NA | 22.5^a^(0-58)m | NA | NA | PCNL 2, URS 5 | NA | NA | Recurrence 1 |
| Rezaee-Zavareh ^23^ | Iran | 2015 | 574 | 1990-2010 | 25 | 4.36% | NA | NA | NA | NA | NA | NA | NA | NA | NA | NA | NA | NA |
| Rifaioglu ^24^ | USA | 2008 | NA | 1997.1-2007.12 | 18 | NA | 48 | 10/8 | 14/4 ^c^ | 13(6-40) | Kidney 11,ureter 7 | 23.1m | NA | NA | URS  8,PCNL 7, spontaneous pass 2,open surgery 1 | NA | No complication | Recurrence 1 |
| Sarier ^25^ | Turkey | 2018 | 3758 | 2009.11-2017.1 | 36^d^ | 0.90%b | 41.6920-63) | 17/5 | 0/22 | 11.6 (4–29) | Kidney 10, ureter 9,urethra 1, bladder 2 | 27.3(3–67)m | NA | Hyperparathyroidism 9,hyperuricaemia 7 | PCNL 1, cystoscopy 3, URS 18 | NA | UTI 1 | No recurrence |
| Sevinc ^26^ | Turkey | 2015 | 897 | 2008.2-2014.12 | 6 | 0.67% | 50.5(31-65) | 3/3 | NA | 10.8 (7.5-22) | Kidney 2, ureter 6 | 28.2 (0-58)m | NA | Ureteral tortuosity  and obstruction 1，Ureterovesical  anastomosobstruction 1 | URS 5,PCNL 1 | Calcium oxalate stone 3, calcium phosphate stone 1, uric acid stone 2 | Hematuria 1 | Recurrence 1 |
| Stravodimos ^27^ | Greece | 2012 | 1525 | 1983.1-2007.3 | 7 | 0.46% | NA | NA | NA | 14.5(4-25) | Kidney 5, ureter 2 | 3.2（2-7）y | NA | NA | PCNL 3, ESWL 3,spontaneous pass 1 | NA | No complication | No recurrence. |
| Streeter ^28^ | UK | 2002 | 1292 | 1975.1-1998.5 | 9 | 0.70% | NA | NA | NA | NA | Kidney 3,ureter 6 | 150^a^ (56–  1280)d | NA | NA | Nephrostomy+ESWL 1,PCNL 3,open surgery 1, nephrectomy 1, Conservative treatment 3 | NA | 1 graft lost | NA |
| Verrier ^29^ | France | 2012 | 3000^f^ | 1978-2010 | 31 | 0.90%^e^ | 40.5(19-68) | NA | 30/1 | NA | Kidney 11,  ureter 16  bladder 4 | <3 months in 4 kidney-graft lithiasis,  8.5 years (8-14) in 27 posttransplantation stone | NA | Ureteral obstruction 12, infravesical obstruction 4,UTI 1,donor gifted 4 | Conservative treatment (10stable,2 spontaneous pass) 12,PCNL 2, ESWL 2,open surgery 8, URS 5, cystoscopy 4 | NA | No complication | Bladder stone recurrence 2 |
| Wyatt ^30^ | USA | 2009 | NA | 1982-2005 | 16 | NA | 37.5(8-57) | 6/10 | 12/4 | NA | NA | 7.7y(6m-30y) | NA | Hyperoxaluria 1,  donor gifted 1,  Hyperparathyroidism 7, secondary to infection 3,  Retained foreign body (stent, prolene suture) 6 | PCNL 16 | Struvite stone 3,Calcium oxalate stone 4,Uric acid stone 1,  Calcium phosphate stone 1,  Mixed stone 7 | Residual stone 1 | Recurrence 2. |
| Yigit ^31^ | Turkey | 2004 | 125 | 1999-2003 | 3 | 1.60%b | 32.3(18-53) | 1/2 | 3/0 | 5.8 (3-11) | Kidney 1，bladder 2，ureter 2 | 3d,7m,6m | NA | Donor gifted 1,BPH 1, stent unintentionally  left for a long  period 1. | ESWL 1, cystoscopy 1, stent removal 1 | Calcium  oxalate stone 1, infectious stone 2. | No complication | No recurrence |
| Yuan ^32^ | China | 2015 | 1615 | 2000.8-2014.7 | 19 | 1.18% | 38.7(11-65) | 9/10 | 15/4 | 15.2(5-35) | Kidney 9,ureter 9, both ureter and pelvic 1 | 21^a^(3-211)m | Asymptomatic 2,UIT 4, oliguria/anuria 5, dysfuntion 4,hematuria 3,pain 1 | NA | ESWL 5,URS 4,PNCL 6, URS after 2 failure SWL 1,PCNL+URS 1,spontaneous pass 2 | Calcium oxalate stone 7,uric acid stone 2,mixed stone 2,struvite stone 3 | No complication | No currence |

ARF, acute renal failure;UTI, urinary tract infection; ESWL, extracorporeal shockwave lithotripsy; PCNL, percutaneous nephrolithotomy;PCN, percutaneous nephrostomy; URS, ureteroscope; NA, not available; BPH, benign prostate hyperplasia; d, day; m, month; y, year

^a^ Median

^b^ Excluding bladder stone.

c Including an autotransplant.

^d^ 36 were diagnosed with graft urolithiasis at follow-up. But only 22 patients with minimally invasive surgical procedures were included.

^e^ Including double kidney, 102 pancreas-kidney and 118 liver-kidney transplantations.
